# A Dynamic Analysis of IRS-PKR Signaling in Liver Cells: A Discrete Modeling Approach

**DOI:** 10.1371/journal.pone.0008040

**Published:** 2009-12-01

**Authors:** Ming Wu, Xuerui Yang, Christina Chan

**Affiliations:** 1 Department of Computer Science and Engineering, Michigan State University, East Lansing, Michigan, United States of America; 2 Department of Chemical Engineering and Material Science, Michigan State University, East Lansing, Michigan, United States of America; 3 Department of Biochemistry and Molecular Biology, Michigan State University, East Lansing, Michigan, United States of America; University of California San Diego, United States of America

## Abstract

A major challenge in systems biology is to develop a detailed dynamic understanding of the functions and behaviors in a particular cellular system, which depends on the elements and their inter-relationships in a specific network. Computational modeling plays an integral part in the study of network dynamics and uncovering the underlying mechanisms. Here we proposed a systematic approach that incorporates discrete dynamic modeling and experimental data to reconstruct a phenotype-specific network of cell signaling. A dynamic analysis of the insulin signaling system in liver cells provides a proof-of-concept application of the proposed methodology. Our group recently identified that double-stranded RNA-dependent protein kinase (PKR) plays an important role in the insulin signaling network. The dynamic behavior of the insulin signaling network is tuned by a variety of feedback pathways, many of which have the potential to cross talk with PKR. Given the complexity of insulin signaling, it is inefficient to experimentally test all possible interactions in the network to determine which pathways are functioning in our cell system. Our discrete dynamic model provides an *in silico* model framework that integrates potential interactions and assesses the contributions of the various interactions on the dynamic behavior of the signaling network. Simulations with the model generated testable hypothesis on the response of the network upon perturbation, which were experimentally evaluated to identify the pathways that function in our particular liver cell system. The modeling in combination with the experimental results enhanced our understanding of the insulin signaling dynamics and aided in generating a context-specific signaling network.

## Introduction

A major challenge in current molecular biology is to understand the dynamic behavior of biological systems. Biological processes consist of many interacting components, exceeding the human capacity to systematically analyze them, thus requiring methods to reduce the complexity and thereby enhance their accessibility [1]. Thus, a central idea in systems biology is to construct models to help reveal the design principles of biological systems [2]. Over the past decade researchers have successfully identified genes and proteins involved in many different signaling processes and assembled them into pathways and networks. However, to use these interaction maps to develop a detailed dynamic understanding of the functions and behaviors that are specific to a biological system has yet to be realized. Typically the pathways, networks and interaction maps in the literature or databases are obtained from different cellular systems and conditions, and may not be applicable to all systems and under all conditions. Therefore it is unclear which pathways are relevant to a particular system that is under investigation.

Here we proposed a systematic, dynamic analysis approach that reconstructs a phenotype-specific network of cell signaling. As an example, insulin signaling, a well-studied and complicated signaling network, in mammalian cells is composed of branched downstream signaling pathways and various feedback mechanisms, which could benefit from modeling. In a separate study, our group identified the involvement of a novel player, PKR, in the insulin signaling network of HepG2 cells [3]. Previously known as an immune response protein, PKR was found to be affected by insulin, and more importantly, PKR modulated the upstream mediators of insulin signaling, creating a feedback loop [3]. Considering the complexity of this insulin-PKR signaling network, we applied modeling to unravel a context-specific, insulin-PKR-signaling network, relevant to HepG2 cells, to obtain a better understanding of the role of PKR in this complex signaling network, and further to aid hypothesis-driven research on the system dynamics.

A dynamic model that can efficiently integrate the literature knowledge and experimental data [4] could provide valuable working hypothesis to guide the experimental investigation [5], [6]. Discrepancies arising between the simulation and experiment could indicate potential missing or erroneous links that may lead to new discoveries. In general, the approaches used to model the dynamics of a biochemical reaction may be broadly classified into three categories, stochastic, kinetic, and discrete modeling. Stochastic modeling captures the detailed reacting process of each molecule in the system [7], kinetic modeling focuses on the concentration change of the species [2], while discrete modeling describes the general profile of a process evolving rather than the time course of the molecules or species concentration. A major advantage of discrete dynamic models, unlike detailed kinetic or stochastic models, is that they do not require a complete set of explicit biochemical or kinetic parameters in order to capture the system dynamics [8]. This information is difficult to obtain for most biological signaling processes [9], primarily because of the diversity and complexity of the signaling network, the incomplete knowledge of the regulatory mechanisms, and the lack of quantitative time-series data [10]. Thus, discrete dynamic modeling provides an alternative modeling approach, to test or perturb a hypothetical regulatory network, *in silico*, to assess for consistencies in the information accumulated on a particular system by different experimenters under different conditions. [8].

### Discrete Dynamic Modeling in Molecular Biology

Discrete dynamic or Boolean network modeling applies Boolean algebra to obtain a qualitative, discrete representation of the biological system [11]. A discrete model may associate logical variables with gene expression or protein activity levels, and logical functions with their biological relationships, such as transcription regulation, protein-protein interactions, or biochemical reactions, to characterize the system [9]. With the advent of genome-scale maps of protein-protein interactions and transcription factor-DNA binding data, available from different groups and through databases, such as TRANSPATH (http://www.biobase.de), BioCarta (www.biocarta.com), and STKE (www.stke.org), discrete dynamic models can capitalize upon these resources to derive the network architecture.

Discrete modeling has uncovered many important dynamic features in biological systems. The dynamic model developed by Li et al [12] captured the steady states corresponding to the different biological cell-cycle phases in budding yeast. Further, discrete modeling uncovered robust stationary states corresponding to real biological events in the cell cycle of fission yeast [13], flower morphogenesis [14] and floral cell-fate determination [8] in Arabidopsis, and the segmentation pathways in Drosophila development [15], [16]. Despite these successes in revealing the robust design of biological systems, discrete dynamic simulation is still in its infancy with respect to generating predictions from perturbation experiments. A recent discrete model of Arabidopsis [17] predicted patterns of abscisic acid signal transduction and identified key regulatory components. Here we applied a novel discrete dynamic modeling approach using three-state logic variables, taking into account cell-to-cell variations on the protein activities and reaction rates, and allowing for simulated population effects. As a proof-of-concept application of the discrete dynamic model, we applied the methodology to insulin-PKR signaling in liver cells. The model also provided testable hypothesis that were experimentally validated.

### Insulin Signaling Transduction in Liver Cells

Insulin is one of the major hormones controlling the complex hepatic metabolic responses. Insulin binds to the insulin receptor tyrosine kinase (IR), which recruits and phosphorylates the insulin receptor substrate (IRS) proteins at tyrosine residues [18], [19]. Upon tyrosine phosphorylation, the IRS proteins initiate, through different binding mechanisms [20], various downstream signal transduction cascades, including c-Jun N-terminal kinase (JNK) [21], [22] and phosphoinositide 3-kinase (PI3K) [23], which in turn catalyze the formation of the lipid second messenger phosphatidylinositol-3,4,5-triphosphoate (PIP3). PIP3 may initiate the downstream Akt/protein kinase B (Akt/PKB) [24], and atypical protein kinase C (aPKC) [25]. In addition, IR can also phosphorylate the SH2 domain of Shp2 protein, a tyrosine phosphatase, and the SH3 domain of its adaptor molecule Grb2. Activated Grb2 recruits Sos1 which, in turn, activates the MAPK pathway mediated by the Ras protein.

Insulin signaling is tuned at the IRS level, by a large number of regulators [26], [27]. The activity of IRS is largely regulated by its phosphorylation at the different residues. Tyrosine phosphorylation of IRS facilitates the recruitment of IRS substrates and promotes insulin signaling [22], while the serine phosphorylations generally suppress the activities of IRS by blocking the interaction between IRS and IR [28], inhibiting the tyrosine phosphorylation of IRS [29], or inducing the degradation of IRS [30]. A number of serine residues have been identified to negatively regulate the activity of IRS1, in particular, Ser307 (equivalent to Ser312 in human IRS1). A number of signaling molecules, such as IκB kinase (IKK) [31], mammalian target of rapamycin (mTOR) [32], PKCζ [33], S6 Kinase 1 (S6K1) [34], ERK [35] and JNK [36], [37] have been shown to function as IRS serine kinases that induce insulin resistance by promoting the inhibitory phosphorylation of IRS (reviewed in [26], [38]). Research in our group has shown that as one of the downstream target of insulin signaling, PKR is intricately involved in regulating the insulin signaling process through a feedback mechanism. The phosphorylaton of PKR is down-regulated by insulin through the IRS-PI3K-AKT-PP1 pathway, and PKR inhibits insulin signaling by promoting the phosphorylation of IRS at Ser312, through IRS serine kinases such as IKK and JNK [3].

Given the complexity of insulin signaling, it is inefficient to experimentally test all possible interactions in the network to confirm their functionality. Thus, we proposed a novel discrete dynamic network model that integrates potential interactions and components of insulin signaling and its feedbacks in HepG2 cells. Our simulations provided patterns of dynamic activation of each component and generated testable hypothesis on the response of the network upon perturbation, which were used to direct the experiments.

## Methods

### Assumptions of Discrete Dynamic Model

#### 1. Network architecture defines the major dynamic characteristics

The dynamic behaviors are determined primarily by the network architecture. This assumption is based on the observation that biological systems tend to maintain their functionality despite environmental and intrinsic noise that cause fluctuations in their protein/RNA levels or reaction rates [39]. Many kinetic models based on differential equations show stable patterns under a wide range of kinetic parameters, thus, we hypothesize that the higher level abstraction of coarse-grained discrete models can also successfully recover the major patterns of the system. For example, a network of segment polarity regulation in Drosophila development modeled by kinetic gene interactions [40], was subsequently also successfully modeled using a Boolean network model [15]. The Boolean model indeed was able to reproduce the major patterns captured with a numerical method approach, and correctly predicted some of the ectopic patterns from the perturbation data (i.e., over-expression and mutant studies). Similarly, differential equation- [41] and logic-based [42] methods successfully modeled mammalian cell cycle progression with a network consisting of the same central components. The logic model was able to capture the dynamic features of the system. These examples suggest that in many cases the dynamic behavior of biological systems rely strongly on the network architecture and more subtly on the kinetic parameters.

#### 2. Discrete variables can represent the activity level of the network components

In Boolean networks, the binary on/off representations of mRNA or protein level, and the logical functions for the interactions can be directly derived from qualitative experimental data of activating/inhibitory role of the various elements in the network. Since the switching behavior (i.e., activation/inhibition) is quick in many signal transduction processes, it is reasonable to approximate the actual continuous process of the state of the network components with discrete levels [9].

#### 3. Modularity

Given that modularity is a design principle of biological systems [43], isolated or individual modules of the system should be able to provide insight into the mechanisms that govern the systems behavior or to mechanistically explain a particular dynamic behavior of a system [14]. Therefore, we assume that we do not need the entire network to gain mechanistic insights. To investigate the role of PKR in insulin signaling of liver cells, we include pathways mediated by IKK, JNK and ERK because they have the potential, according to the literature or our experiments, to mediate the interaction between PKR and IRS (insulin signaling). We do not consider many of the other factors or pathways involved in insulin signal, which may be regulated by IKK, JNK or ERK but do not interact with PKR or feedback to IRS. The same holds for the other intermediates (i.e., Raf, ShGS, etc.). Our results show that this network module is able to provide insight into the mechanisms that govern the system dynamics.

#### 4. The building of the signaling network

**Figure 1:**
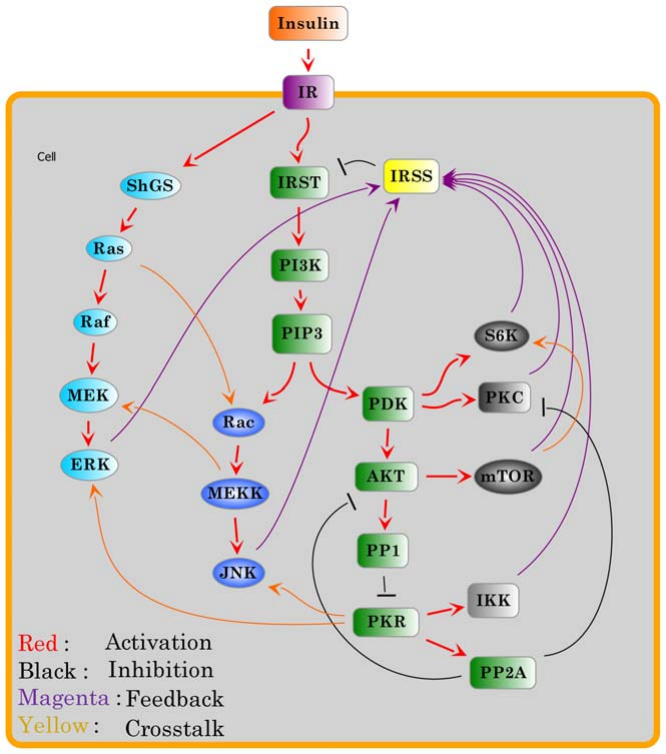
Signaling network of insulin signaling and its feedbacks. Each arc is assigned an attribute—either activation or inhibition.

The signaling network can be formalized in terms of an oriented graph, where the vertices represent the elementary components involved in the process and the arcs describe the regulatory interactions between those components. In the signaling network, each directed arc reflects the direction of information flow from the source vertex to its target in the signal transduction, and is labeled with a positive or negative sign which defines activation or inhibition, respectively. The network in Figure 1 was drawn with the JDesigner in the SBW toolbox (www.systembiology.org).

#### 5. The definition of the variables and initial states

We represent the cell variation by the fluctuations in their initial protein activity levels, which we assume to be a normal distribution around a control state for each protein. We associate each vertex in the signaling network with a discrete variable, which has three states representing the activity of the protein----0: lower than control, 1: the control state, 2: higher than control. Thus by definition every component starts at the “control” state in the absence of insulin stimulation. We use a discrete model with variables and transition rules (see below) to represent the dynamic profile of a cell and we sample a large population of cells. We choose an initial state for each regulatory component from a distribution centered at the “control” state, which reflects the cell-to-cell variation in the protein levels or activities. The variation captured by the distribution represents the degree of stochastic noise in the protein activities.

#### 6. The definition of perturbation and noise

Constraints are assigned to components to mimic the perturbations on the network. If a protein is constantly inhibited (in an experiment), we restrain the state of its corresponding variable in the model to be always in either 0 (lower than control) or 1 (control state). Since we apply a distribution on the initial states of the protein activity for describing the cell-to-cell variation, we can use the variation over the distribution of initial states to describe the degree of noise that causes the cell-to-cell variation in the protein activities. Variation in the cellular protein activity can lead to cell-to-cell variation [44] that contributes, in part, to the noise in the system. Thus, the model without any noise will start at the “control state” for every protein and for each cell, whereas large amount of noise or variation in the protein activity would manifest in many of the proteins not being at their “control state” and varying from cell to cell.

#### 7. The specification of the state transition

We define transition rules based on the activation/inhibition attributes on the arcs in the signaling network. Two operations: shift up and shift down adapted from the triple logic are applied in the model (see Table 1). If an activator is in a state “higher than control”, (e.g. the kinase phosphorylation is increased), the state of its target will be shifted upwards. In contrast, if the state of an inhibitor is higher than control, its target will be shifted downwards in the next updating event. For some components, there may be multiple regulators that are active in one updating event and the combinatory effect is determined by comparing the number of activating to inhibitory factors. The target is shift-up if there are more activating factors, and vice versa [12], [13]. If the number of activating and inhibiting factors equal, we assume the target remains at the control or current state. Finally, the state of a component will decay if its regulators can no longer maintained their active state.

**Table 1:** Shift up and shift down operations.

Shift up		Shift down	
Current-State	Updated-State	Current-State	Updated-State
0	1	0	0
1	2	1	0
2	2	2	1

**Figure 2:**
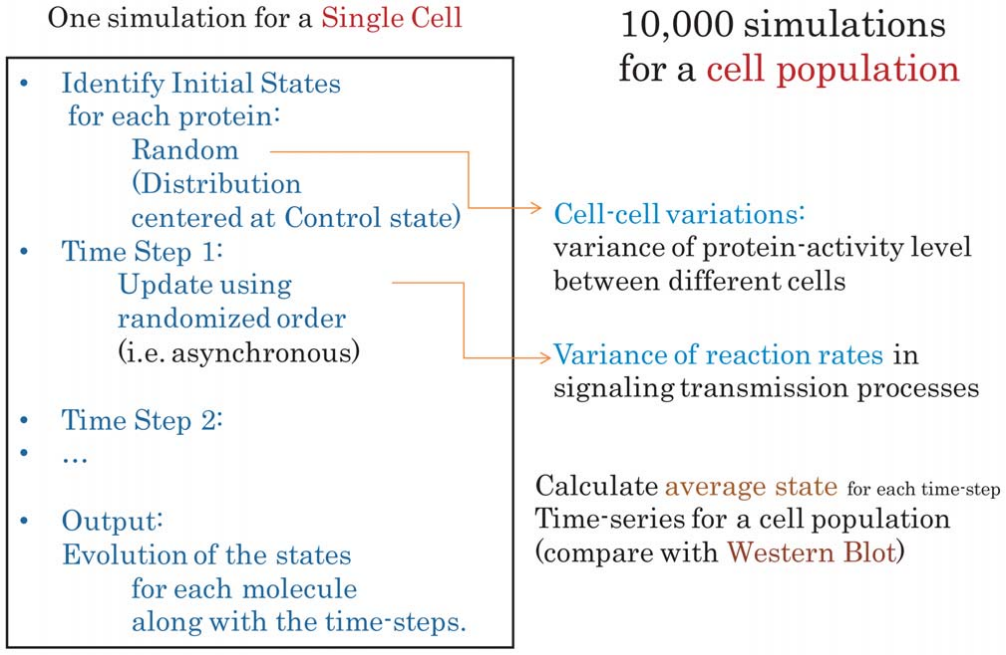
The simulation process of our discrete dynamic model.

In the simulation each run starts with its own set of randomly generated initial states and a simulation result represents the dynamic profile of a single cell in the population. By assuming that cells response independently to a signal, we can simulate a large number of independent runs to mimic a population effect and measure the average evolving profile for the population (see Figure 2).

Time is modeled by regular intervals called time-steps. Since most components in our network are kinases or phosphotases, and most reactions are protein phosphorylation and dephosphorylation, we assume that the duration of the activation/inhibitions and the decay processes in the signaling transduction are comparable and approximated by one time-step. Since the reaction rates may be different from cell to cell even for the same interaction, we apply asynchronous updating of the state, which is realized immediately, rather than renewing every variable simultaneously at each time-step. Thus, the relative rates of the different reactions can be specified by the ordering of the update, which implies that, although the response may be similar, the rate of response varies from cell to cell. The rules for an asynchronous algorithm can be written as: 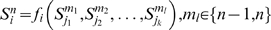 where 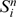 is the state of component 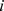 at time-step 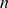, and 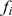 is the transition function associated with 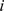 and its regulators 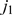 to 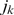, and the time-point corresponding to the last change of the regulators can be either the last or current round of updates.

Since we embed the uncertainties in the signaling process by applying random initial states and the asynchronous updating, we need a population large enough to obtain a stable dynamic profile. As shown in the graph (Figure S1), in a small population the dynamic profile varies in different simulations due to the embedded randomness, but in a large population the profile is stable. Such behavior has been observed in real biological systems where a single cell exhibits randomness while a large colony shows an inherent and specific dynamics [45], [46].

The dynamic model was implemented by custom MATLAB code, with a random-order asynchronous updating algorithm, which updates each component, one by one, in a randomized order at each time-step, in each cell. The order in which the components are updated is randomly chosen from a uniform distribution over all possible permutations, to achieve random timing for each regulatory interaction. As an example, let us consider a single interaction: component A activates component B. Suppose A and B are both in 1, the control state, but at the next time-step a stimulus activates A and shifts its state to 2. If applying synchronous updating, the state of B at this “next time-step” is 1 (since it is based on the current state of A, which is 1), so B will be activated in the following step, thus the time required for the activation from A to B is fixed to one time-step. In asynchronous updating, the state of B on the next time-step is either 1 or 2 depending on whether the updating of B is prior to the updating of A, which is randomly chosen in a cell, thus the timing of the activation is variable. The state-change process of this example is shown in Figure S2.

### Experimental Materials and Methods

#### 1. Cell culture and reagents

Human hepatoblastoma cells (HepG2/C3A) were cultured in Dulbecco's Modified Eagle Medium (DMEM) (Invitrogen, Carlsbad, CA) with 10% fetal bovine serum (FBS) (Biomeda Corp, Foster City, CA) and penicillin-streptomycin (penicillin: 10,000 U/ml, streptomycin: 10,000 µg/ml) (Invitrogen, Carlsbad, CA). Freshly trypsinized HepG2 cells were suspended at 5×105 cells/ml in standard HepG2 culture medium and seeded at a density of 106 cells per well in standard six-well tissue culture plates. After seeding, the cells were incubated at 37°C in a 90% air/10% CO2 atmosphere, and two milliliter of fresh medium was supplied every other day to the cultures after removal of the supernatant. The HepG2 cells were cultured in standard medium for 5–6 days to achieve 90% confluent before any treatment. Human insulin was purchased from Sigma-Aldrich (St. Louis, MO), okadaic acid (OA), IKK inhibitor (SC-514), JNK inhibitor (SP600125), and ERK inhibitor (PD 98059) from EMD Biosciences (San Diego, CA).

#### 2. Insulin treatment

Human insulin was stocked in HEPES buffer, which was therefore used in controls for all the experiments with insulin treatment. We treated the cells with insulin at concentrations lower than 1 nM to mimic the physiological concentrations [47]. At 95% confluence, cells were deprived of serum for 16 hours prior to each experiment and subjected to insulin treatment for the indicated doses and times at 37°C in serum-free medium.

#### 3. Western blot analysis and immunoprecipitation

HepG2 cells were lysed as described previously [48]. Total protein levels were quantified by BCA assay kit from Pierce Biotechnology Inc (Rockford, IL). 20–40 µg of total protein were resolved by SDS-PAGE gels from Bio-Rad (Hercules, CA), transferred to nitrocellulose membranes, and probed with primary and secondary antibodies. Biotinylated protein ladders (Cell Signaling, Beverly, MA) were loaded to one well of each SDS-PAGE gel, and anti-biotin antibody was used to detect the protein ladders on Western blots. Antibody detection was performed using the enhanced chemiluminescence kit from Pierce Biotechnology and imaged on the Molecular Imager ChemiDoc XRS System from Bio-Rad. Immunoprecipitation was performed as described previously [48]. The western blots were quantified using the Quantity One software (Bio-Rad). Phospho site-specific anti-IRS1 (Tyr941), anti-Akt (Thr308), and anti-Akt were purchased from Abcam (Cambridge, MA), phospho site-specific anti-IRS1 (Ser312), PKR (Thr451), anti-IRS1, anti-PKR, and anti-beta actin antibodies from Sigma-Aldrich. Secondary anti-rabbit and anti-mouse antibodies were purchased from Pierce Biotechnology Inc.

## Results

We collected and integrated the literature information on insulin signaling with emphasis on the different feedback pathways and crosstalk with PKR and built a consensus regulatory network that contained all the components and potential interactions (see Method 1 and Figure 1). We formularized the signaling process based on the network architecture into a discrete dynamic model (see Method). By combining model simulations and experimental validations, we identified the essential components and pathways that function in our specific cell system.

Each simulation represented a single cell and we simulated a large population to obtain an average pattern of the dynamic activation of each component in the network, with or without insulin stimulation. We compared the simulated and the experimental profiles of IRS tyrosine, IRS serine, and PKR phosphorylation levels.

**Figure 3:**
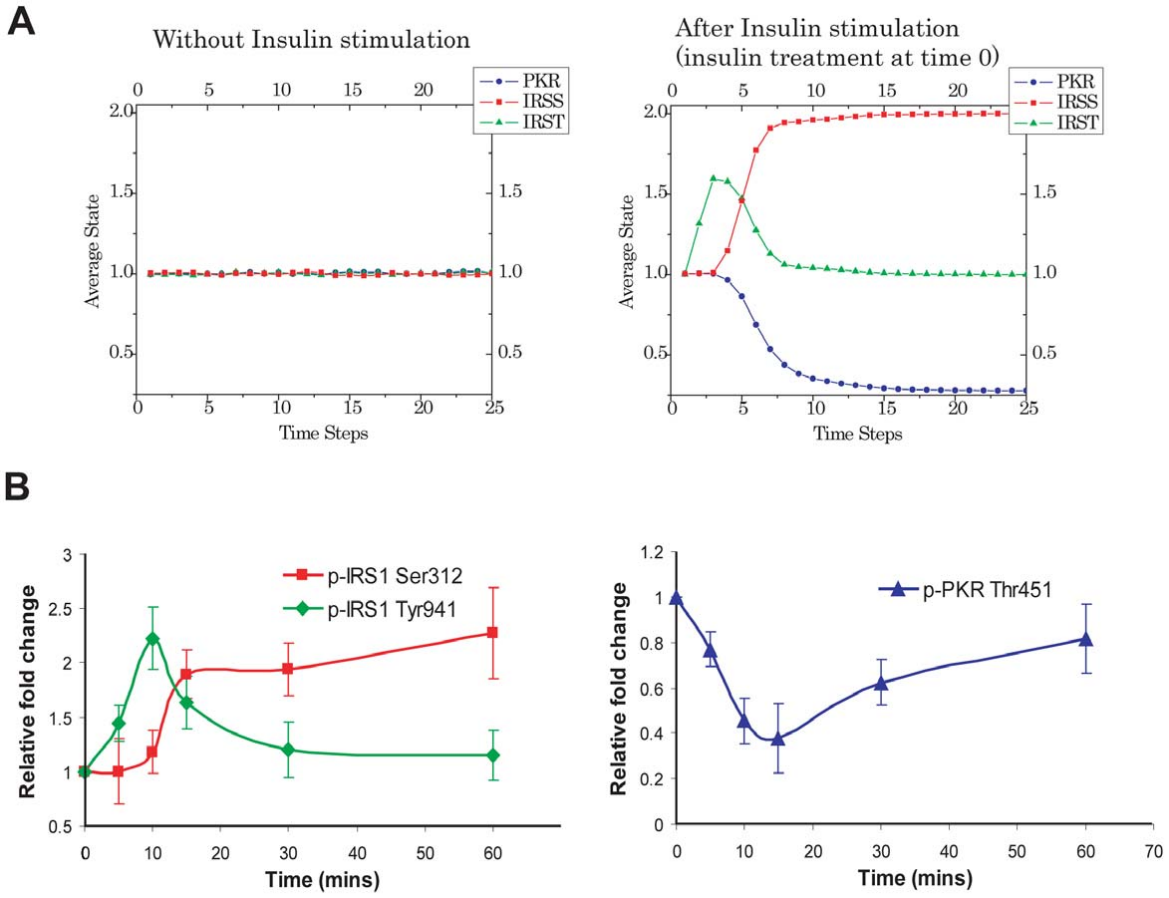
The simulated and experimental results of the PKR and IRS phosphorylation. A) Model simulations with or without insulin stimulation. The simulation is on the initial model including the potential interactions and components from the literature and our experiments. The interactions emphasized are the level of IRS serine phosphorylation (IRSS), IRS tyrosine phosphorylation (IRST), and the PKR phosphorylation. B) Time series of the PKR and IRS phosphorylation upon insulin stimulation at time 0. HepG2 cells were exposed to 1 nM of insulin for 5, 10, 15, 30, or 60 minutes. After treatment, the cells were harvested, and western blot analysis was performed to detect the total and phosphorylated levels of PKR and IRS1 [3]. The phosphorylation levels of PKR (blue) and IRS1 (red for p-IRS1 at Ser312 and green for p-IRS1 at Tyr941) were quantified and normalized to the total protein levels of PKR and IRS1, respectively, and are expressed as the average of four samples±SD from four independent experiments.

The simulation result suggests plausible dynamic profiles of the interacting network upon insulin stimulation, and based upon the components and interactions potentially involved in our particular cell system (Figure 1, see Introduction: Insulin signaling transduction in liver cells). The IRS and PKR activity are maintained in a stationary state (control state) prior to insulin stimulation. Upon insulin stimulation, the amount of tyrosine phosphorylated IRS protein increases, followed by a decrease in the level of PKR phosphorylation and the accumulation of the serine phosphorylated IRS protein. After some time, tyrosine phosphorylated IRS downregulates or returns to basal levels, while the serine phosphorylation of IRS remains elevated (Figure 3A).

### Identification of Essential Pathways

#### Breakdown of the Akt-PKR loop: PP2A-Akt positive feedback may not be involved

We designed several western blot experiments to measure the changes in IRS tyrosine, IRS serine, and PKR phosphorylation levels in response to insulin in liver cells. The experimental results (Figure 3B) show an initial increase in the IRS tyrosine phosphorylation upon insulin stimulation, which is subsequently followed by an increase in IRS serine phosphorylation accompanied with a decrease in IRS tyrosine phosphorylation, likely due to the negative feedback within the signaling network. This agrees with the predictions by the dynamic model.

However, from the experiments, we observed that the PKR phosphorylation decreases with the IRS tyrosine phosphorylation, but rather than remaining at a constant, low level as predicted by our model, PKR phosphorylation actually increases 15 minutes after insulin stimulation (Figure 3B). Such discrepancy indicates potentially incorrect connections in the network. Since the network is built upon the current information obtained from different experimental conditions and different groups, certain interactions may not necessary be involved in or contribute to the dynamics of our system.

**Figure 4:**
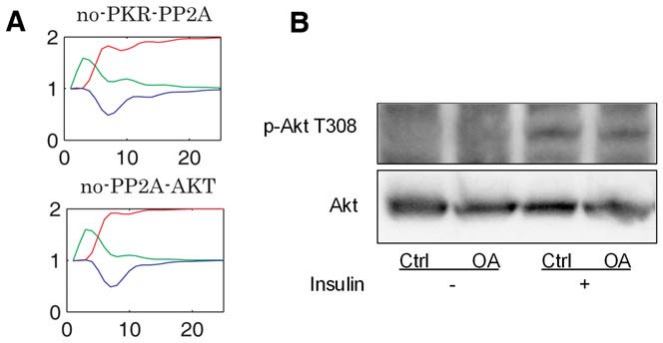
Breakdown of the Akt-PKR loop. A) *In silico* knock-out. For each subgraph, an interaction is deleted from the model and the simulation is performed on the knock-out model. The interaction being knocked out is labeled at the top of each subgraph in the form of “no”-regulator-target. Red line: IRS serine phosphorylation, green line: IRS tyrosine phosphorylation, blue line: PKR phosphorylation. B) Western blot: effect of PP2A inhibitor (OA, 2 nM [56]) on the phosphorylation of Akt at Thr308. OA: Okadaic Acid.

We then examined the contribution of each regulatory interaction through *in silico* knockout experiments (Figure 4A and Figure S3). Biologically “knockout” means removing or mutating a particular gene in the genome to shut down its expression, and thus one can investigate the outcomes to identify the functional role of the gene that is knocked out. Here we borrow the concept of “knockout” to describe our *in silico* experiments of deleting a certain interaction to access its contribution on the dynamics of the system. Thus, in our model, by removing the interaction, we are suggesting that the pathway is not active in our system. Further, it is not always necessary to knockout the activity of a target to assess its role in a pathway; reducing the activity can be sufficient to access the functional contribution of an interaction and to experimentally evaluate whether or not a prediction is valid. It appears that by either deleting the PP2A-AKT or PKR-PP2A pathway, the PKR phosphorylation profile then agrees with the experimental observation. Further investigation into the literature on the network architecture identified a local positive feedback module involving AKT-PP1-PKR-PP2A (Figure S4) [3], [49]–[52]. The existence of such a local module would help to maintain a continuous (persistent) activation of AKT, once stimulated, which would lead to a continuous inhibition of the PKR signal. Therefore, for the model to match the experimental results, this positive feedback loop should not be active in the system. From the literature, PKR is known to phosphorylate B56α, the regulatory subunit of protein phosphatase 2A (PP2A), which then activates the catalytic subunit of PP2A [51]. Indeed, previously we showed that PKR induces the phosphatase activity of PP2A in HepG2 cells [3]. Therefore the PKR-PP2A pathway should be present in the network and the PP2A-Akt pathway should be excluded in order to match the experimental dynamic profile of the PKR phosphorylation in response to insulin.

It has been shown in different systems, but not in HepG2 cells, that PP2A can induce the dephosphorylation of Akt at amino acid residue Thr308, and thereby suppress the activity of Akt [53]–[55]. Therefore, we tested whether PP2A exploits any regulation on Akt phosphorylation in HepG2 cells. Okadaic acid (OA) [56], a specific PP2A inhibitor, with or without the simulation of insulin, did not affect the phosphorylation of Akt at Thr 308 (Figure 4B). This indicates that in our cell system, HepG2 cells, in response to 1 nM insulin, PP2A does not dephosphorylate Akt. Therefore, the feedback from PP2A to Akt does not play a role in the signaling network of HepG2 cells, which confirms the model prediction.

#### ERK feedback does not play a significant role

The *in silico* knock-out results (Figure S3) show that many of the interactions, especially the feedbacks through the IRS serine phosphorylation, such as mTOR-IRSS, S6K-IRSS, IKK-IRSS, JNK-IRSS, ERK-IRS, if deleted, have no significant affect on the dynamics of IRS tyrosine/serine and PKR phosphorylation levels. This suggests these feedbacks may be redundant in the system. Since we are trying to elucidate the regulatory role of our novel component, PKR, in the insulin signaling network, we focused on the IKK, JNK, and ERK mediated feedbacks, which directly interact with PKR.

**Figure 5:**
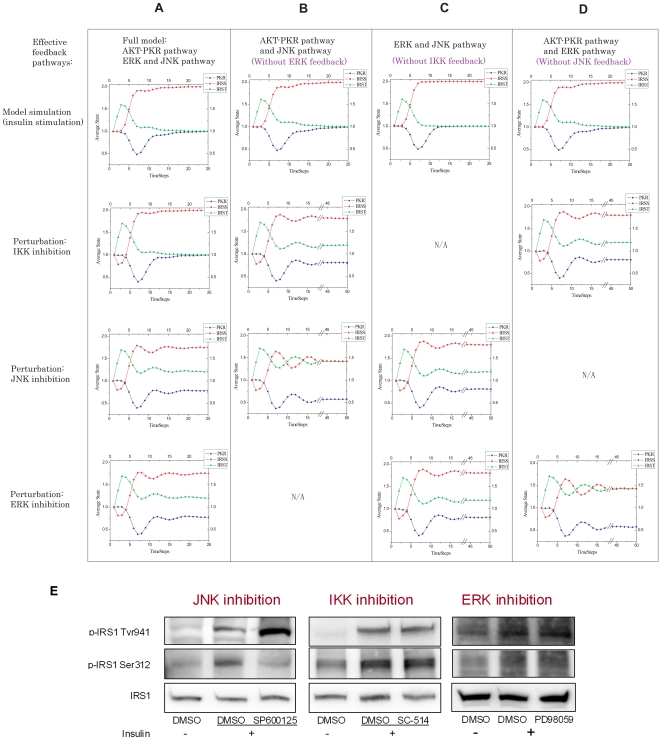
Identification of essential feedback pathways through *in silico* perturbation study. Each column represents a different architecture containing. A) Original ERK+JNK+IKK feedback pathways, B) Only with IKK+JNK feedback pathways, C) Only with ERK+JNK feedback pathways, and D) Only with ERK+IKK feedback pathways. Row 1 the model simulation is performed without any perturbations. Row 2 the model simulation is performed on IKK inhibition. Row 3 the model simulation is performed on JNK inhibition. Row 4 the model simulation is performed on ERK inhibition. E) Western blot: effects of JNK, IKK and ERK inhibitors on the phosphorylation of IRS1 upon simulation with 1 nM insulin for 15 mins.

In order to elucidate the contribution of the different feedback pathways, we evaluated topologies that contained only two of the three pathways, JNK+IKK, ERK+JNK, and ERK+IKK, in addition to the original topology containing all the ERK, JNK, and IKK feedbacks (Figure 5A–D) to better assess which of these kinases plays a role, and hence is non-redundant in our cell system. We performed *in silico* perturbation studies on these 4 different network architectures upon inhibition of JNK, IKK, or ERK, and obtained the dynamic profiles of the IRS tyrosine/serine and PKR phosphorylation levels, which was compared with the experimental results upon inhibition of IKK, JNK, or ERK (Figure 5E).

The experimental result shows the JNK inhibitor significantly down-regulated the IRS serine phosphorylation and up-regulated the IRS tyrosine phosphorylation, as compared with the normal, unperturbed insulin stimulated network. Therefore, the model without the JNK feedback (Figure 5D) is incorrect. However, the IKK or ERK inhibitor does not have a significant effect on the phosphorylations of IRS (Figure 5E). This is inconsistent with the IRS phosphorylation profiles under JNK, IKK and ERK inhibition predicted by the model containing all three pathways, where the JNK and ERK inhibition have similar profiles (Figure 5A). Therefore, we hypothesize that all three feedbacks are not essential in the regulation of the IRS-PKR system.

Among the three single-elimination models, removing the ERK pathway (JNK+IKK) provided the most significant change in the dynamic profiles of IRS phosphorylation upon JNK inhibition as compared with that of the IKK inhibition (Figure 5B), as suggested by the experimental results (Figure 5E, JNK vs. IKK inhibition). Thus we hypothesize that the ERK feedback should not be included in our insulin signaling network, which we confirmed experimentally (Figure 5E, ERK inhibition). Although the experimental results suggest that the IKK inhibition does not affect the phosphorylation of IRS1 (Figure 5E), the simulation suggests that removing the IKK feedback from the network would give similar results upon JNK and ERK inhibition (Figure 5C), which does not match the experimental results (Figure 5E). Thus, we performed further perturbations to assess whether the IKK is an essential pathway in the network.

#### Application of the simulation model in PKR over-expression

**Figure 6:**
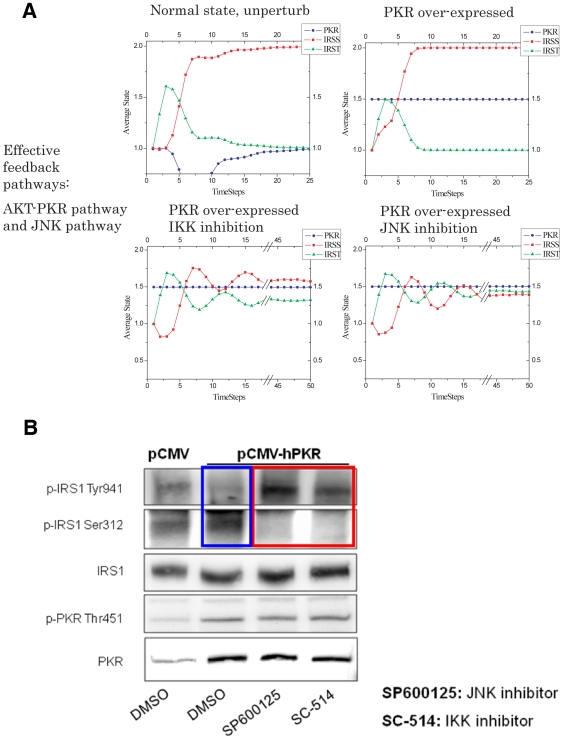
Application of the simulation model in PKR over-expression. A) *In silico* perturbation. Simulation of PKR over-expression and IKK or JNK inhibition with PKR over-expressed. Simulations are based upon the model including the JNK and IKK pathways but without the ERK feedback. B) Western blot results of the tyrosine and serine phosphorylation of IRS1 and the phosphorylation of PKR, after 15 mins of 1 nM insulin treatment, when PKR is over-expressed in HepG2 cells, with or without the IKK/JNK inhibition. IKK and JNK restores Tyr and reduces Ser phosphorylation of IRS1 (red box), as compared to the control with PKR over-expressed (blue box).

**Figure 7:**
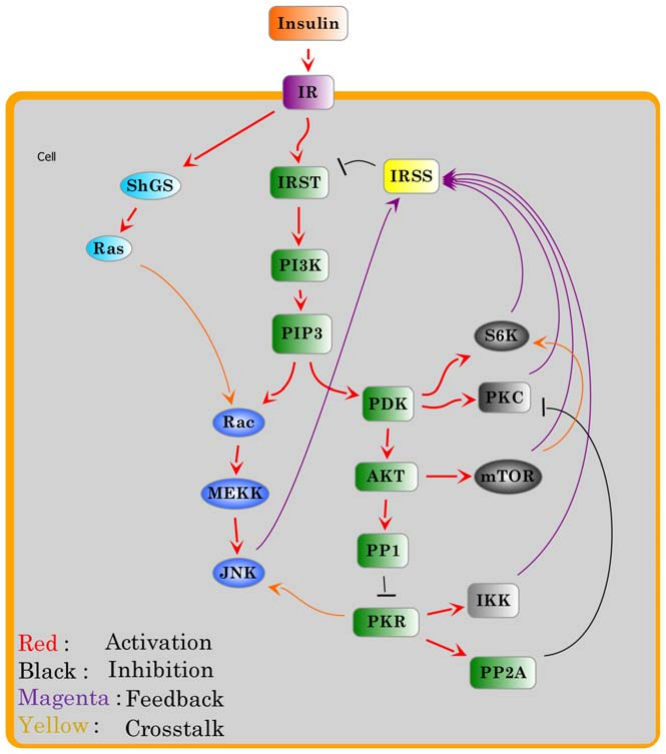
Essential signaling network of insulin signaling and its feedbacks. The direct interaction between PP2A and AKT is removed. The ERK feedback is removed, together with the downstream factors in the ERK pathway that do not have an effect on the regulation of insulin signaling.

To further evaluate which pathways are essential in the dynamic model, we simulated the case of PKR over-expression with the network topology model without the ERK feedback to serine phosphorylation of IRS. Figure 6A shows that PKR over-expression increased the Ser phosphorylation and decreased the Tyr phosphorylation of IRS1, which is supported by the experiments (Figure 6B comparing lanes 1 and 2). Next we simulated the inhibition of IKK or JNK upon PKR-over-expression in the model, and found that in both cases, the Ser phosphorylation of IRS1 is decreased while the Tyr phosphorylation was increased (Figure 6A). These model predictions matched the experimental observations (Figure 6B). As expected, inhibition (Figure 5) or silencing of ERK (Figure S5) does not have any effect on the Ser and Tyr phosphorylations of IRS1. Taken together, these results confirm the model predictions and support the ability of the discrete dynamic model to predict and guide experimental analyses. Interestingly, by comparing the IKK inhibition (in the model and experiments) in the PKR over-expression case, we found that IKK mediates the effects of PKR on IRS1 phosphorylation, however, the basal level of IKK, if not activated, does not play a significant role in regulating IRS1 phosphorylation. Therefore, the essential pathways involving IRS-PKR signaling in our cell system is shown in Figure 7.

### Robustness of the Signaling Network

**Figure 8:**
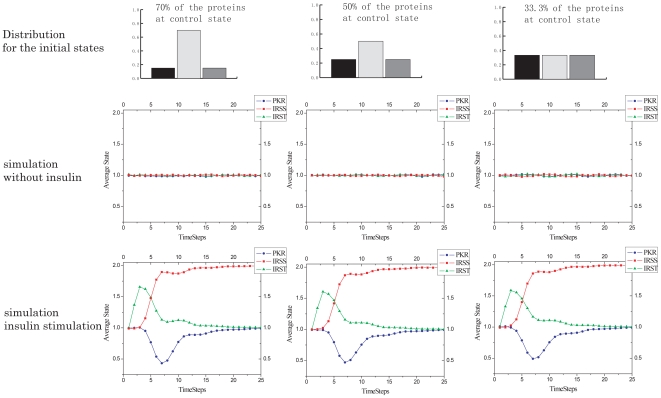
Robust dynamics against noise. In row 1 the noise is represented by the variations over the distribution of the initial states of each component. Simulation without or with stimulus are shown in row 2 and row 3, respectively. In column 1, 70% of the initial protein activity levels are at the control state, indicating minor noise; in column 2, 50% are at the control state; and in column 2, the components are equally likely to be assigned to any of the three states, indicating a higher level of noise. Simulations are based upon the essential pathway model that excludes the ERK pathway. Colors on the distribution: light grey: control state (1), dark grey: higher than control (2), black: lower than control (0).

We used the distribution of initial states to describe the cell-to-cell variation of protein activity levels due to environmental or endogenous noise. The variation over the distribution of initial states represents the degree of noise. Multiple simulations were run on the architecture consisting of the essential pathways, under varying levels of noise, with and without insulin stimulation. The simulation results (Figure 8) suggest the dynamics of insulin signaling are very stable against the noise associated with variation in the distribution of initial states or cell-to-cell variation. Such robustness may arise from the multiple negative feedbacks within the network, that help maintain a very stable, stationary state in the absence of stimulation, and ensure specific signaling patterns despite the uncertainties involved.

We further simulated the perturbation models under a different noise level (e.g., uniform distributed initial states) and found the dynamic profiles to be robust (Figure S6) and almost identical to the previous simulation results where most (70%) of the cells initiated from the control state. Thus, it is not likely that the dynamic features discovered are artifacts of the modeling methods. The robustness of the signaling network suggests the dynamics are encoded in the architecture of the insulin-signaling network within the cell system, and such robustness in the dynamics ensures a robust transduction of insulin signaling.

## Discussion

We enhanced the discrete modeling approach by extending the on/off logic of traditional discrete Boolean models to a three-level logic model with “high, control, low”. Also, rather than assigning ad-hoc absolute values as initial states, for each component we defined its state prior to stimulation by a distribution centered on the “control state” and compared the effect of the perturbation with the control state, and thereby avoiding the problem of defining the initial state in traditional Boolean network models.

In contrast to the traditional Boolean models that apply one model to describe the whole process, we apply an individual model to every single cell with each cell having a distinct initial state from a distribution centered on the control state. The simulation produces a response by obtaining the average effect of a large group of cells. This modeling approach takes into account the population effect and cell-to-cell variation, such that the resultant model can capture the deviations or alterations from the control state that is conceptually consistent with the western blot experiments.

### Limitations in Discrete Modeling

#### Lack of exact timing

Discrete dynamic models do not represent or capture the exact timing, but the resultant dynamic patterns provide a dynamic profile evolving with “time-steps”, which may contain artifacts due to the network reconstruction and the updating rules at each time step. Previous biological Boolean network models were simulated in such a way that all the components changed their states simultaneously by one unit of time, based on the assumption that every reaction in the network takes exactly one unit of time in the signaling process. However, in a real biological system, the reactions are not homogenous, different reactions may have different rates, thus “synchronous” updating may not be appropriate. Klemm et al. [57] evaluated the stability of attractors in Boolean networks and found that many attractors disappear with slight perturbation in the time-steps, suggesting that they may be artifacts due to the synchronous updating.

Asynchronous updating has been suggested to reduce the artifacts due to the assumption of uniformity in all the reactions arising from synchronous updating [42], [58]. In asynchronous clocking, we update the state of each component one by one at each time-step, and any state update is realized immediately and will affect the other components' state change, even within the same time-step. In this way, some of the cells realize the updated state immediately while other cells take longer to respond and therefore do not update at the same time, thus leading to variable reaction times/rates for each cell. We implemented asynchronous updating in the modeling method (see Method) by randomly generating the reaction order for each time step and in each simulation. This allows us to take into account the uncertainty of the reaction rates in different cells.

Even with asynchronous updating, there still may be artifacts that arise due to the discrete timing, such as small fluctuations. For example, a simple regulatory network with only three components is shown in the Figure S7, where the simulation of a qualitative model is compared with the result of a kinetic model. Figure S7 also shows that the artifacts in the synchronous updating would significantly affect the correct prediction of the system dynamics.

#### Loss of subtle dynamics

Discrete models are based solely on the qualitative relationships of the network elements, and thus may lose certain subtle dynamics. It has been shown that in some cases certain kinetic parameters are essential for a system's behavior (e.g., the reaction constants of phosphatases determine the response time and duration under weak stimulation in the MAPK kinase cascade model in [59]), which cannot be captured in qualitative models. The discrete models are essentially simplifications of the kinetic equations in that the reaction laws are reduced to a non-parametric form [60]. For example, the actual reaction “A→B with kinetic constant k” may be considered in the qualitative representation as “if A is high, B could be up-regulated”, but the effect of parameter “k” is neglected. Thus the series of bifurcations (or switch-like behavior) that depend on the parameters may (or may not) be captured as patterns of state-change in discrete models, the latter provides only a rough approximation of the real reaction system [10].

Since our insulin signaling network is somewhat homogenous with only protein phosphorylations/dephosphorylations, our model treats every interaction and feedback the same, and does not take into account the different strength or time scale of the interactions and feedbacks, which are possible in real systems, especially in heterogeneous networks with both protein interactions and transcription regulations. Nevertheless, as long as the quantitative experimental data are available that captures such differences, we can further adjust the model to integrate such information, by increasing the discrete levels and applying more subtle rules. For example, one can apply longer updating time for transcription regulations than for protein interactions.

### Advantages of Discrete Modeling

The advantage of discrete modeling is that only qualitative information is required. Kinetic modeling (and stochastic modeling) requires detailed kinetic parameters and initial concentrations for each component in the network, which is usually not available for all the components in the network. Without the need for kinetic parameters, the discrete modeling approach can model and simulate larger networks, given the abundant qualitative interaction maps of biological systems that are available.

Discrete dynamic modeling represents a higher abstraction of biological network as compared with kinetic modeling, and perturbations on the structure of the network are more easily simulated with the discrete models. Kinetic approaches usually perturb parameters of a system rather than the structure, because a different set of kinetic parameters (and even different equations) may be required if the network structure is changed. Therefore, it is very difficult to obtain those parameters for the perturbed system, despite the availability of experimental data.

As a simplified qualitative model, discrete dynamic model has finite system-states, thus one can analyze the design principle of the network structure by exploring all the possible states to find the stationary ones [12]. Similar analysis is usually very difficult with kinetic or stochastic models because the parameter space is too large to sample thoroughly.

In conclusion, our novel modeling approach provides a systematic description of the biological process, which enables testable predictions that can serve as working hypothesis for experimental evaluation. By combing modeling and experiments, we can develop a hypothesis-driven framework that can be iteratively refined to enhance our understanding of the insulin signaling network dynamics in the context of a particular cell system. Such a framework that relies solely on the network architecture can be easily extended to other dynamic network systems and serves as a basis to guide model-based experiments and potentially more detailed inquiry into the regulatory mechanisms of biological networks.

## Supporting Information

Figure S1 With a small population the dynamic profile varies significantly in the different simulations due to the embedded randomness, whereas in a large population the profile is stable.
(0.38 MB TIF)

Figure S2 An example of the implementation of synchronous and asynchronous updating for a cell.
(0.16 MB TIF)

Figure S3 In silico knock-out. For each subgraph, an interaction is deleted from the model and the simulation is performed on the knock-out model. The interactions being knocked out is labeled at the top of each subgraph in the form of “no”-regulator-target. Red line: IRS serine phosphorylation, green line: IRS tyrosine phosphorylation, blue line: PKR phosphorylation.
(0.44 MB TIF)

Figure S4 A local positive feedback module from AKT to PP2A
(0.07 MB TIF)

Figure S5 The effects of ERK silencing or inhibition on the phosphorylation of IRS1 in PKR over-expressed cells. (A) Reverse transfection of suspended HepG2 cells were performed with scrambled siRNA (NC) or siRNAs of ERK1 and ERK2 together for 24 hours and the transfected cells were cultured in regular media for another 24 hours. Next, the forward transfection of empty vector pCMV6-XL5 (pCMV6) or plasmid containing PKR cDNA sequence (pCMV6-hPKR) was performed, followed by the treatment of insulin (0.5 nM) for 15 minutes. After treatments, cells were then harvested and western blot analysis was performed to detect the protein level of ERK, and total and phosphorylated levels of PKR and IRS1. (B) In HepG2 cells, the forward transfection of empty vector pCMV6-XL5 (pCMV6) or plasmid containing PKR cDNA sequence (pCMV6-hPKR) was performed and the cells were then treated with the pharmaceutical inhibitor of ERK, PD98059 (PD98059, 50 uM) or DMSO, vehicle of PD98059, for 1 hour, followed by the treatment of insulin (0.5 nM) for 15 minutes. After treatments, cells were then harvested and western blot analysis was performed to detect the total and phosphorylated levels of IRS1 and PKR.
(0.40 MB TIF)

Figure S6 Robust dynamics against noise. The noise is represented by the variation in the distribution of initial states for each component. Colors on the distribution: light grey: control state (1), dark grey: higher than control (2), black: lower than control (0). Perturbations and simulations are based upon the essential pathway model that excludes the ERK pathway, with uniform distributed initial state for each component.
(0.29 MB TIF)

Figure S7 A small regulatory network with catalytic activation/inhibition. S is the stimulator (input) of the system. Upon stimulation of S, A activates B. There is a negative feedback from B to A. Simulation results of a kinetic model, a qualitative (Boolean) model with asynchronous or synchronous updating are shown.
(0.23 MB TIF)
