# Supplementary figures and images for: A Dynamic Analysis of IRS-PKR Signaling in Liver Cells: A Discrete Modeling Approach
Source: PLoS One. 2009 Dec 1;4(12):e8040. doi: 10.1371/journal.pone.0008040 (PMC2779448; doi:10.1371/journal.pone.0008040)

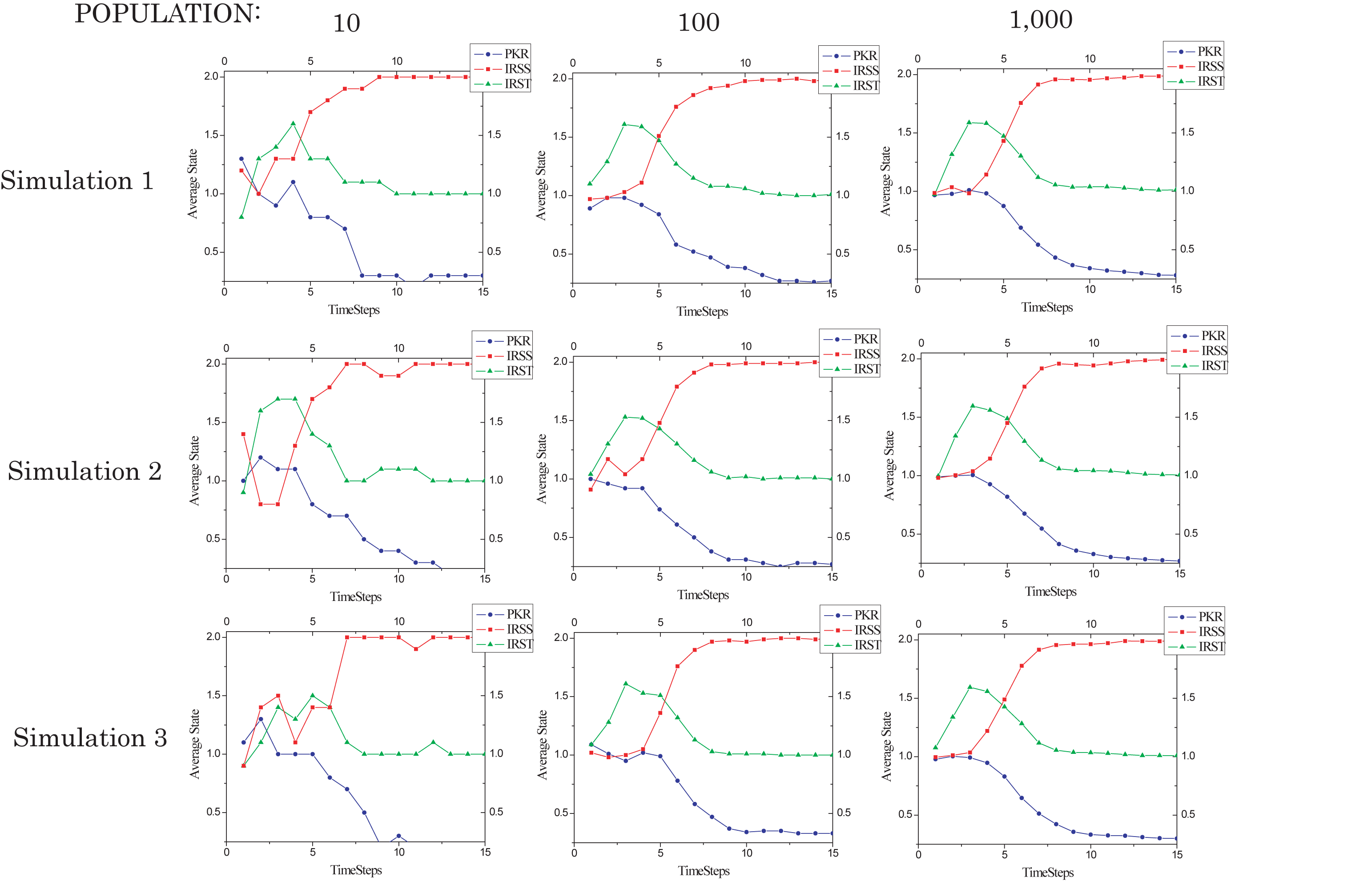

Supplement: Figure S1 — With a small population the dynamic profile varies significantly in the different simulations due to the embedded randomness, whereas in a large population the profile is stable. (0.38 MB TIF) [file pone.0008040.s001.tif]

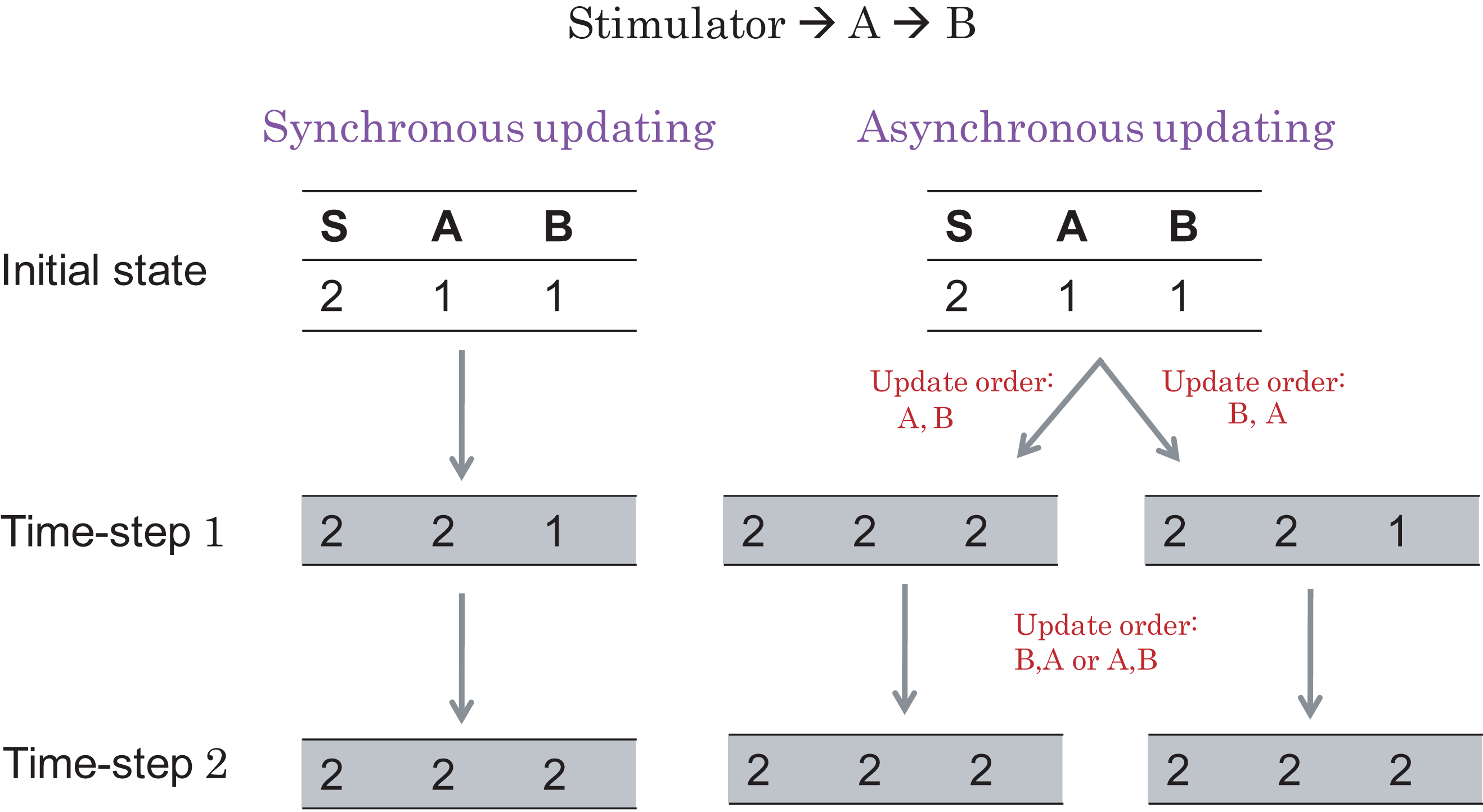

Supplement: Figure S2 — An example of the implementation of synchronous and asynchronous updating for a cell. (0.16 MB TIF) [file pone.0008040.s002.tif]

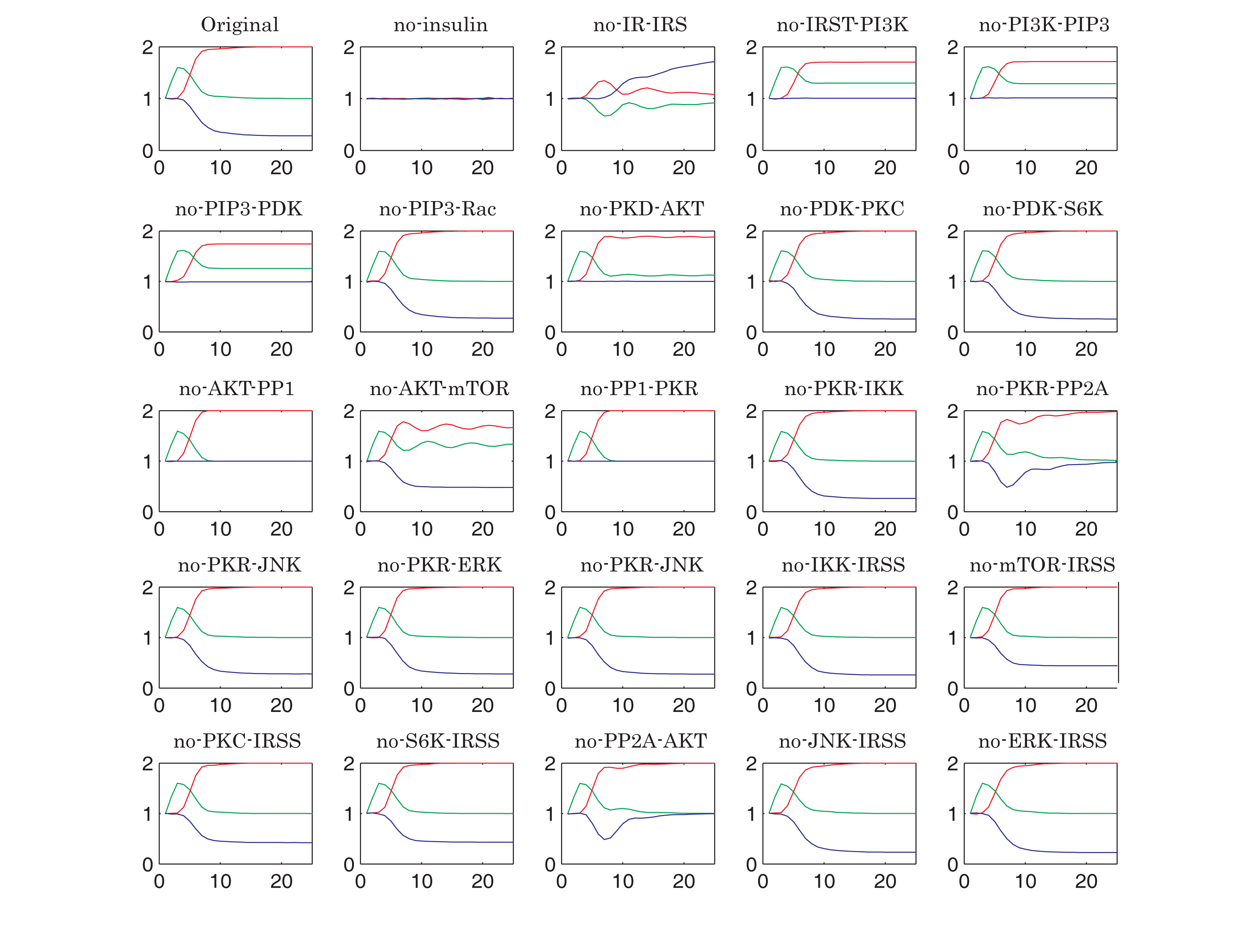

Supplement: Figure S3 — In silico knock-out. For each subgraph, an interaction is deleted from the model and the simulation is performed on the knock-out model. The interactions being knocked out is labeled at the top of each subgraph in the form of “no”-regulator-target. Red line: IRS serine phosphorylation, green line: IRS tyrosine phosphorylation, blue line: PKR phosphorylation. (0.44 MB TIF) [file pone.0008040.s003.tif]

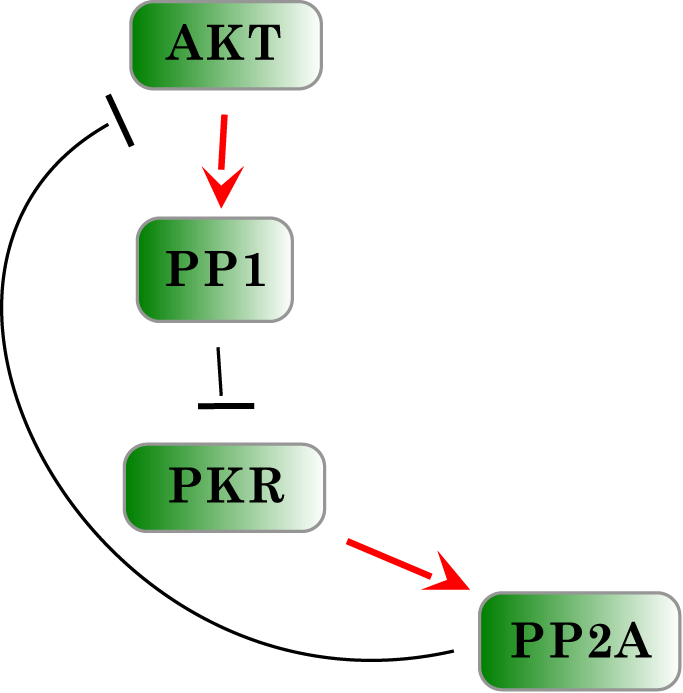

Supplement: Figure S4 — A local positive feedback module from AKT to PP2A (0.07 MB TIF) [file pone.0008040.s004.tif]

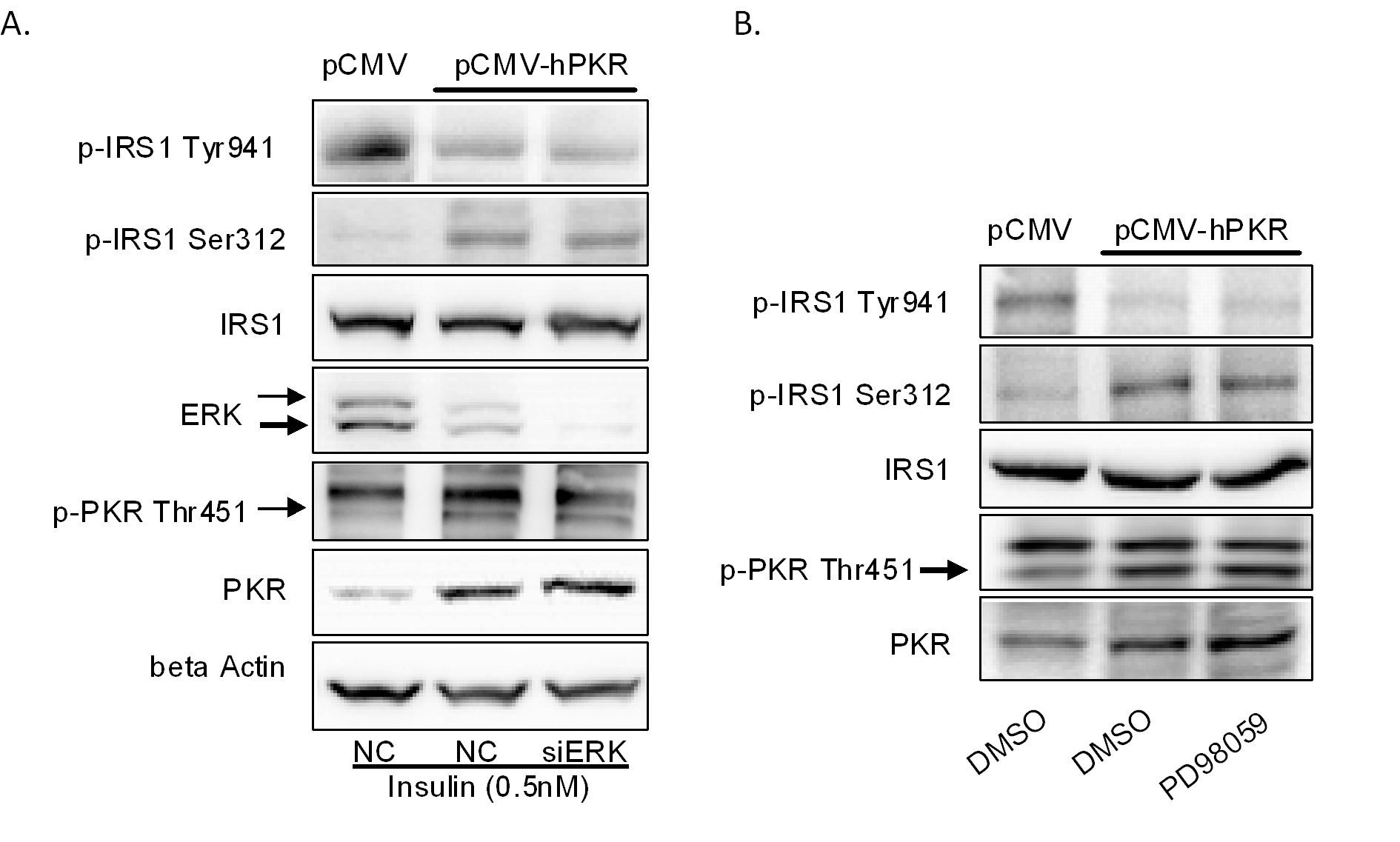

Supplement: Figure S5 — The effects of ERK silencing or inhibition on the phosphorylation of IRS1 in PKR over-expressed cells. (A) Reverse transfection of suspended HepG2 cells were performed with scrambled siRNA (NC) or siRNAs of ERK1 and ERK2 together for 24 hours and the transfected cells were cultured in regular media for another 24 hours. Next, the forward transfection of empty vector pCMV6-XL5 (pCMV6) or plasmid containing PKR cDNA sequence (pCMV6-hPKR) was performed, followed by the treatment of insulin (0.5 nM) for 15 minutes. After treatments, cells were then harvested and western blot analysis was performed to detect the protein level of ERK, and total and phosphorylated levels of PKR and IRS1. (B) In HepG2 cells, the forward transfection of empty vector pCMV6-XL5 (pCMV6) or plasmid containing PKR cDNA sequence (pCMV6-hPKR) was performed and the cells were then treated with the pharmaceutical inhibitor of ERK, PD98059 (PD98059, 50 uM) or DMSO, vehicle of PD98059, for 1 hour, followed by the treatment of insulin (0.5 nM) for 15 minutes. After treatments, cells were then harvested and western blot analysis was performed to detect the total and phosphorylated levels of IRS1 and PKR. (0.40 MB TIF) [file pone.0008040.s005.tif]

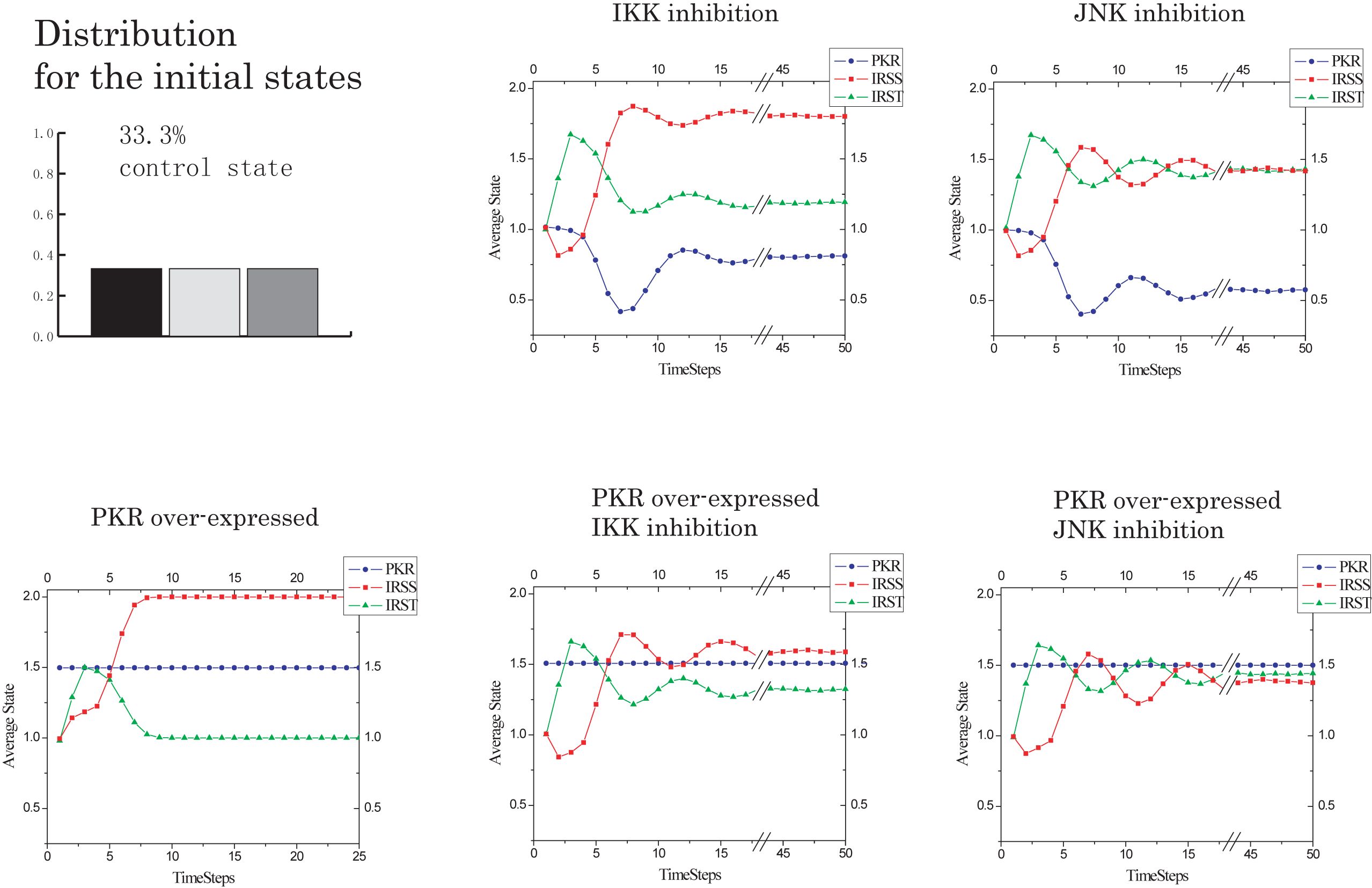

Supplement: Figure S6 — Robust dynamics against noise. The noise is represented by the variation in the distribution of initial states for each component. Colors on the distribution: light grey: control state (1), dark grey: higher than control (2), black: lower than control (0). Perturbations and simulations are based upon the essential pathway model that excludes the ERK pathway, with uniform distributed initial state for each component. (0.29 MB TIF) [file pone.0008040.s006.tif]

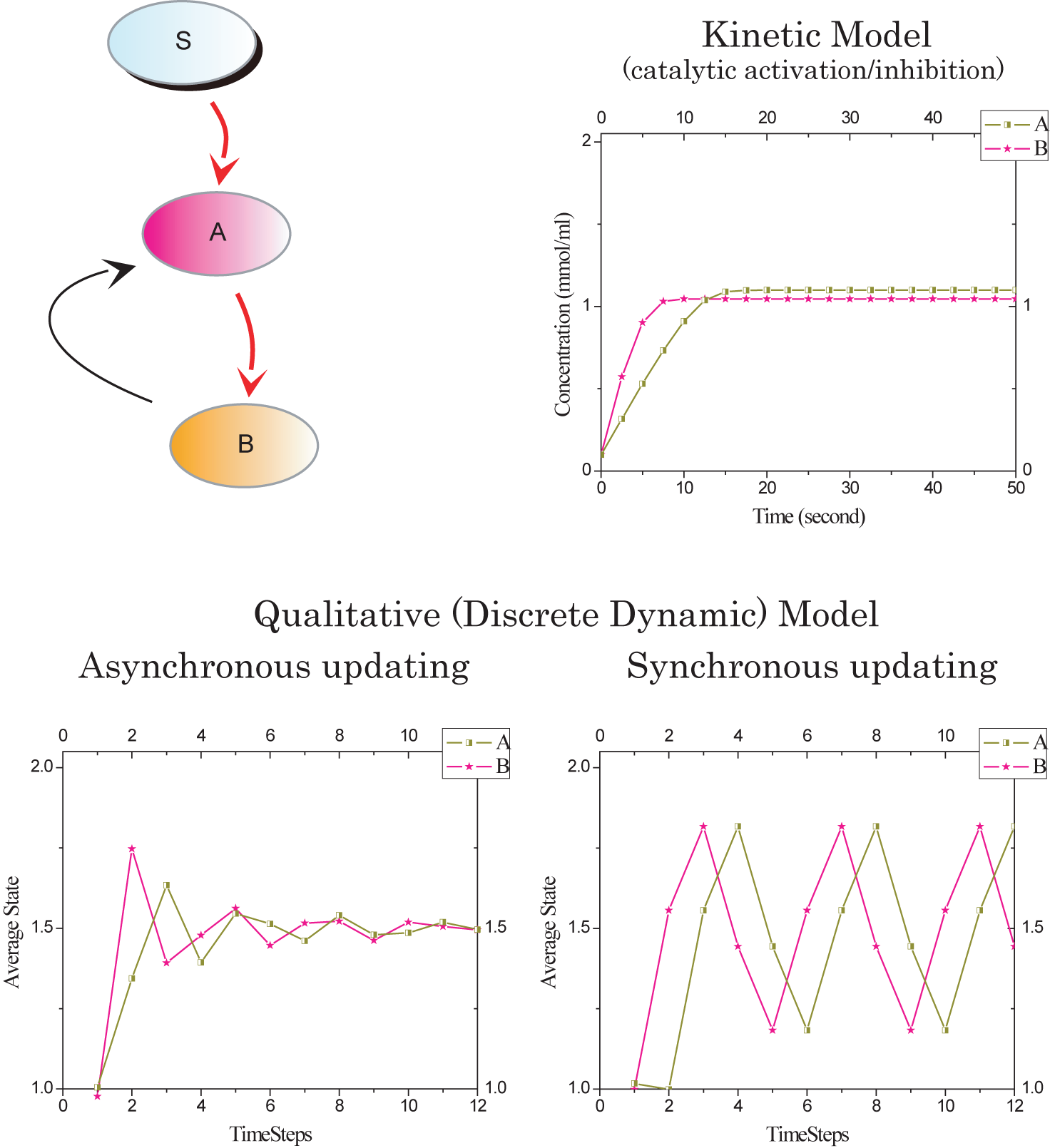

Supplement: Figure S7 — A small regulatory network with catalytic activation/inhibition. S is the stimulator (input) of the system. Upon stimulation of S, A activates B. There is a negative feedback from B to A. Simulation results of a kinetic model, a qualitative (Boolean) model with asynchronous or synchronous updating are shown. (0.23 MB TIF) [file pone.0008040.s007.tif]
